# Determinants of Motivation among Healthcare Workers in the East African Community between 2009–2019: A Systematic Review

**DOI:** 10.3390/healthcare8020164

**Published:** 2020-06-10

**Authors:** Rose Nabi Deborah Karimi Muthuri, Flavia Senkubuge, Charles Hongoro

**Affiliations:** 1 School of Health Systems and Public Health (SHSPH), Faculty of Health Sciences, University of Pretoria, Pretoria 0028, Gauteng Province, South Africa; flavia.senkubuge@up.ac.za (F.S.); chongoro@hsrc.ac.za (C.H.); 2 Developmental, Capable and Ethical State Division, Human Sciences Research Council (HSRC), Pretoria 0001, Gauteng Province, South Africa; 3 Faculty of Science, Tshwane University of Technology, Pretoria 0183, Gauteng Province, South Africa

**Keywords:** motivation, healthcare workers, health systems, health workforce strengthening, East Africa

## Abstract

Healthcare workers are an essential element in the functionality of the health system. However, the health workforce impact on health systems tends to be overlooked. Countries within the Sub-Saharan region such as the six in the East African Community (EAC) have weak and sub-optimally functioning health systems. As countries globally aim to attain Universal Health Coverage and the Sustainable Development Goal 3, it is crucial that the significant role of the health workforce in this achievement is recognized. In this systematic review, we aimed to synthesise the determinants of motivation as reported by healthcare workers in the EAC between 2009 and 2019. A systematic search was performed using four databases, namely Cochrane library, EBSCOhost, ProQuest and PubMed. The eligible articles were selected and reviewed based on the authors’ selection criteria. A total of 30 studies were eligible for review. All six countries that are part of the EAC were represented in this systematic review. Determinants as reported by healthcare workers in six countries were synthesised. Individual-level-, organizational/structural- and societal-level determinants were reported, thus revealing the roles of the healthcare worker, health facilities and the government in terms of health systems and the community or society at large in promoting healthcare workers’ motivation. Monetary and non-monetary determinants of healthcare workers’ motivation reported are crucial for informing healthcare worker motivation policy and health workforce strengthening in East Africa.

## 1. Introduction

The health workforce is vital to the core functionality of a health system, yet it tends to be overlooked as a key element of upgrading health systems [1]. Health worker shortage is an unfortunately rising phenomenon adversely affecting multiple health systems. There is minimal literature and knowledge on effective strategies on how to combat the hasty deterioration of human resource for health [2], especially in developing countries. More than a decade ago in the World Health Organization (WHO) 2006 report Working Together for Health, 57 countries reported experiencing critical health workforce shortage [3]. By 2018, according to the Global Health Observatory (GHO) by WHO, a global need for 17 million additional skilled health workers was reported [4]. According to WHO, the African region is experiencing both the greatest burden of disease and the lowest density of health workers at 2.2 health workers per 1000 population of healthcare professionals [5]. In the WHO 2006 report, 36 Member States, some of which are part of the East African Community (EAC), were and still are experiencing a critical shortage of human resource for health [3]. Health workforce shortage crisis impedes the performance and quality of healthcare provided, contributing to sub-optimal functionality of health systems. Borghi and colleagues [6] state that the ability of a health system to deliver quality healthcare among the current workforce is partly dependent on healthcare workers’ motivation.

Motivation is a determinant of behaviour characterised by the driving force one has to achieve a certain goal [7]. In the context of health systems strengthening, motivation is one of the driving forces among health workers that can potentially contribute towards the progress of attaining the WHO health system goals, namely improved health level and equity, responsiveness to clients’ non-medical expectations, social and financial risk protection and improved efficiency [8]. According to WHO, motivation is a worthy investment to effectively perform Primary Health Care (PHC) and health workforce strengthening [8,9,10]. The significance of investing in health workforce motivation was affirmed in the Global Conference on Primary Health Care 2018 report [9]. 

Assessing motivation is complex due to various determining factors, both external and internal in nature [6,11,12]. The Hawthorne studies done between 1924 and 1932 by Elton Mayo and Fritz Roethlisberger reported a novel element: that employee motivation was not exclusively monetary-based as previously assumed by employer, employees, researchers and policymakers [13]. Thus, the Hawthorne Effect, which is the overt observation of employee performance resulting in behavioural change such as higher performance [13]. Following the discovery of the Hawthorne Effect, researchers began paying attention to non-monetary motivators [13]. Researchers have found employees are motivated by intrinsic and extrinsic factors [13].

Intrinsic motivation occurs when an individual is driven by interest and satisfaction doing the work itself [14]. On the other hand, extrinsic motivation is when an individual is driven by the external consequences of performing a task [14]. There are multiple theories on human motivation, with the most common being Maslow’s Hierarchy of Needs. Nonetheless, three theories, namely the Two-Factor theory by Herzberg, Cognitive Evaluation Theory by Porter and Lawler and Self-determination Theory by Ryan and Deci [15], similarly view motivation from the perspective of intrinsic and extrinsic factors or motivators. 

In this study, we aimed to do a systematic review, to consolidate the existing evidence on the determinants of motivation among healthcare workers in the East African Community (EAC). The EAC consists of six countries, namely the Republic of Burundi, Republic of Kenya, Republic of Rwanda, Republic of South Sudan, Republic of Uganda and United Republic of Tanzania, which are connected by a common integration agenda [16]. The EAC countries are either low- or lower-middle-income developing countries, with most populations living in the rural areas. The EAC state health systems have been described as resource-limited settings [17,18,19,20], severely weakened [21] and fragile [22]. There are various studies that have been done about healthcare workers’ motivation. To date, there is no recent systematic review that has been done on determinants of healthcare workers’ motivation in the East African Community between 2009 and 2019. Using a narrative synthesis approach, we report on significant intrinsic and extrinsic determinants of motivation among healthcare workers in East Africa in the last 10 years (2009–2019). 

This review answers the question, ‘What are the determinants of healthcare workers’ motivation in the East African Community between 2009 and 2019?’ The present review informs researchers what has been done in this area while simultaneously identifying the gaps in knowledge pertaining to this area of study. Healthcare workers will gain knowledge on the determinants of motivation among themselves and their colleagues within the health sector. Health policymakers and implementers will be able to use this information to create evidence-based policies to enhance healthcare workers’ motivation, thus progressively contributing to health systems strengthening among the countries within the East African Community. 

## 2. Materials and Methods 

The current systematic review selection process was guided by the Preferred Reporting Items for Systematic Review and Meta-Analyses (PRISMA) statement [23]. In addition, the Risk for Bias in Systematic Reviews (ROBIS) tool [24] was used to perform quality assessment in the present systematic review. We objectively used the ROBIS [24] and the PRISMA [23] tools to guide and refine our review process. This was important to ensure our systematic review addressed the research question while maintaining sound methodological and synthesis quality. 

### 2.1. Selection Criteria

The inclusion and exclusion criteria were applied at all stages of title, abstract and full-text screening. The inclusion criteria included original, peer-reviewed studies on motivation among healthcare workers in the East African Community, studies published in the English language and published between 2009 and 2019. Healthcare workers in any sector of health such as public, private-not-for-profit (including faith-based health facilities), and private-for-profit-sector health facilities were included in the review. 

The exclusion criteria comprise grey literature, reviews, non-peer-reviewed literature and studies not published between 2009–2019. Studies published on healthcare workers’ motivation but in other continents other than Africa, specifically countries that are not part of the EAC, were excluded. In addition, studies published in any other language but English and studies published on motivation among workers or students other than healthcare workers were excluded. Finally, studies that assess/measure other constructs but report it as motivation were excluded. 

**Figure 1:**
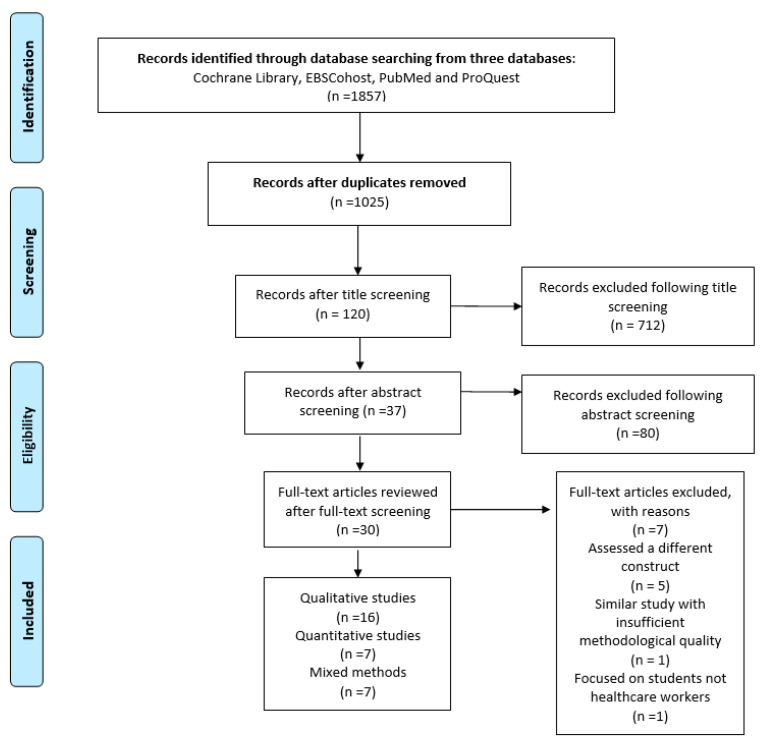
The Preferred Reporting Items for Systematic Review and Meta-Analyses (PRISMA) flow diagram on healthcare workers’ determinants of motivation in the East African Community (EAC), 2009–2019.

The PRISMA flowchart is an illustration of the number of articles included and excluded during the entire selection process, in line with the PRISMA statement [23] (see Figure 1). 

### 2.2. Data Sources and Search Strategy

The systematic search for relevant articles was done using four databases namely, Cochrane Library, PubMed, EBSCOhost and ProQuest. The search terms were related to “motivation”, “healthcare workers”, “healthcare professionals” and “Africa” and were adapted appropriately across the databases. Boolean operators that were used to enable a comprehensive search were “AND” and “OR”. The limitations included the English language and peer-reviewed articles published between 2009 and 2019. 

### 2.3. Data Extraction

Studies retrieved from the three databases were exported to a referencing manager software called Endnote X8^®^ (Clarivate Analytics, Philadelphia, PA, United States of America). On Endnote X8^®^, the duplicates were identified and eliminated. This was followed by title then abstract screening processes. The review team did the full-text screening. The entire screening process was guided by the selection criteria. The number of studies included and excluded are documented and illustrated in the PRISMA flow diagram [23]. The studies that failed to meet the inclusion criteria at the full-text screening phase were documented with reasons.

### 2.4. Risk for Bias

In this systematic review, risk for bias assessment was performed using the Joanna Briggs Institute (JBI) Critical Appraisal Checklist tools for quantitative and qualitative studies included [25]. For mixed methods studies, the Mixed Methods Appraisal Tool (MMAT) [26], was used for assessment of the same. We performed quality assessments according to the study design of each study reviewed. The results of the appraisals are reported in the methodological quality section. 

### 2.5. Data Analysis

Data synthesis and tabulation were utilised to capture and convey the determinants of healthcare workers’ motivation in this review. From the selected studies, the following were captured: author(s) name(s), year of publication, country of study, aim of the study, sample size and main findings on the determinants of motivation. Among the main determinants captured, *p*-values were extracted where available. Since this was a narrative review, not a meta-analysis, we used a thematic framework. Thematic framework analysis is a qualitative method that involves selecting, recording and categorizing key issues and themes [27]; in this case, determinants of healthcare workers’ motivation. We thoroughly reviewed each one of the articles using the thematic framework that consisted of familiarization with data, identification, recording, categorization and interpretation [27] of determinants reported into main themes under which sub-themes were presented. The main themes reported were determinants of healthcare workers’ motivation at the individual, societal/cultural and organizational/structural levels. Under the three main themes, we further categorised data into sub-themes, namely motivators and demotivators. Motivators versus demotivators and intrinsic versus extrinsic determinants of motivation among the healthcare workers. 

The motivators are facilitators which included determinants that drive the healthcare workers to perform their work better. Demotivators are barriers to motivation and consist of determinants whose presence in the health facilities result in reduction or elimination of motivation. The motivators and demotivators could either be intrinsic or extrinsic determinants of motivation, which were sub-categories in the interpretation of our findings. Healthcare workers’ intrinsic motivational determinants included factors that ignite an individuals’ innate interest and satisfaction to do the work itself [14]. While healthcare workers’ extrinsic motivational determinants involved capturing factors related to an individual being driven by the external values of performing a task [14]. The data collected from each article were presented and summarised in tabular format as per study designs, namely qualitative, quantitative and mixed-method designs; this was for clarity purposes. Subsequently, following the data synthesis we explained the impact of the determinants as pertains to the healthcare workers’ motivation. The main aim of thematic framework analysis is to describe and interpret what is happening in a specific setting [27]; in this case, healthcare workers’ motivation in health facilities. 

## 3. Results

### 3.1. Overview 

The systematic search was performed in four databases, namely Cochrane Library, EBSCohost, PubMed and ProQuest, producing 1857 records. Following exportation of all the records to Endnote X8^®^ citation manager, duplicate records were removed (n = 1025) (as shown in flowchart Figure 1). From the remaining 832 records, title screening was performed, and 712 records were excluded. A total of 120 records were subjected to abstract screening, from which 83 records were excluded. Thirty-seven records were eligible for full-text assessment, and seven records were excluded based on the selection criteria. The reasons for exclusion at the full-text screening stage included studies that assessed a different construct such as satisfaction, efficiency, health system performance and reported motivation (n = 5); a similar study with less detail than the one reviewed with more detail (n = 1); and a study that focused on students, not healthcare workers (n = 1). A total of 30 articles were included in this systematic review. Among the 30 articles reviewed, three research designs were represented, namely 53.3% qualitative studies (n = 16), 23.3% quantitative studies (n = 7) and 23.3% mixed-method studies (n = 7) (see Figure 1 and Table 1, Table 2 and Table 3). 

### 3.2. Characteristics of Included Studies

**Figure 2:**
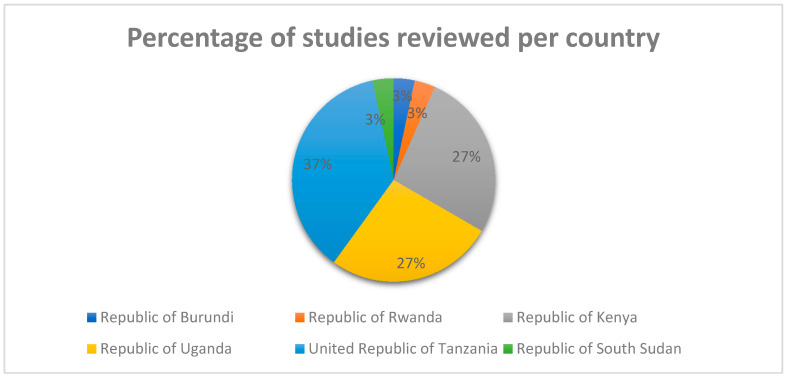
Percentage of studies represented in this review per EAC country.

The studies reviewed focused on the determinants of healthcare workers’ motivation in the EAC. The EAC countries represented in the studies reviewed include the Republic of Burundi (1 study; an equivalent of 3% of the reviewed studies) [45], the Republic of Rwanda (1 study; an equivalent of 3% of the reviewed studies) [36], the Republic of South Sudan (1 study; an equivalent of 3% of the reviewed studies) [43], the Republic of Kenya (8 studies; an equivalent of 27% of the reviewed studies) [11,18,29,33,37,38,44,49], the Republic of Uganda (8 studies; an equivalent of 27% of the reviewed studies) [21,31,34,39,40,46,47,50], and the United Republic of Tanzania (11 studies; an equivalent of 37% of the reviewed studies) [17,19,20,22,28,30,32,35,41,42,48], (see Figure 2). The total number of participants from all the reviewed studies were n = 4358 healthcare workers. 

### 3.3. Individual Level Determinants of Motivation among Healthcare Workers

In this review, individual-level determinants of motivation are primarily intrinsically derived factors within the healthcare worker. At the individual level, six motivators and three demotivators were reported among the reviewed studies.

Altruism, the intrinsic desire to help others (family, community or public) was the most reported individual determinant of motivation among healthcare workers in EAC [11,21,30,35,36,39,42,46]. Attaining professionalism, which is exhibited through professional work ethic, professional attachment and professional pride of being a healthcare worker intrinsically motivated healthcare workers in this review [11,28,50]. An opportunity to learn and seek health-promoting knowledge for personal and community development through receiving education, by being healthcare workers motivated healthcare providers [30,33,40,46,47,49,50]. Work experience was a determinant of motivation, and the number of years in current position as a healthcare worker was a predictor of management and performance aspects [32]. Among community health workers (CHWs), the more experience they gained, the more motivated they reported being [33]. Personal experience with interacting with the health system as a child or because of a family member or an opportunity to be a healthcare provider motivated some healthcare workers because of role models [36,50]. In addition, ease of communication was a motivating determinant of physician and nursing graduates to work in rural areas in Uganda [21].

Demotivating individual-level determinants such as perceived fear of contracting illnesses such as HIV and tuberculosis were reported in a qualitative study [42]. Marital status was another demotivating determinant reported in a quantitative study; specifically, participants who were single or separated reported higher motivation than the ones who were married (p = 0.009) [46]. In Kenya, work conscientiousness was individual-level determinant reported to have decreased following a supervision intervention quantitatively, but the opposite was true qualitatively [37].

### 3.4. Societal/Cultural Level Determinants of Motivation among Healthcare Workers

Societal- or cultural-level determinants of motivation are the social factors where the people within the community play a role in healthcare workers’ motivation. Appreciation, encouragement, support, recognition, respect and admiration (sense of status) from clients, family and the community were some of the societal determinants of healthcare workers’ motivation [11,20,30,33,34,36,40,41]. The positive reception coupled with validation were catalysts in the motivation of healthcare workers to continue working even as volunteers [47]. The opportunity to know, meet and share health-related knowledge and skill with the community, in the health facility and as a mentor, was a motivator of healthcare workers [18,47]. The motivation to know and share knowledge was often explained by a sense of responsibility, desire for healthy behaviour change and commitment to the community and public health [37,42,46]. 

Demotivating societal level determinants reported include challenges of meeting expectations and unrealistic demands of the clients and/or community [11,40]; lack of understanding or awareness of the role of healthcare workers, especially during life-or-death situations [43]; receiving complaints from clients [34]; being disrespected or undervalued by clients and/or community [40,42]; clients reporting being sick following interventions such as family planning (contraceptives) [34]; lack of security and safety [20,21,43], and having small clientele [33]. 

### 3.5. Organizational/Structural Level Determinants of Motivation among Healthcare Workers

In the present review, the organizational-level determinants of healthcare workers’ motivation are related to the organization (the health facility) and the national health systems.

Organizational environment, both social and physical, were reported as determinants of healthcare workers’ motivation in East African studies. With respect to the organizational social environment, specifically healthy professional relationships with colleagues, transformative leaders and supportive committed supervisors and management were some of the determinants of healthcare workers’ motivation [31,33,34,35,37]. An intellectually stimulating environment was an absent and desired determinant of healthcare workers’ motivation in health facilities [18,21]. The organization (health facility) physical environment or rather the lack of a properly operational physical environment, characterised by no clean water, no electricity and poor furniture, among other things, were demotivators reported in various studies [17,22,35,43,49].

Workload was a determinant of healthcare workers’ motivation. Manageability of workload resulted in higher motivation [38]; however, the opposite is also true. Researchers found that heavy, unmanageable workload was reported in various health facilities [21,22,42,43,48]. The heavy workload led to pressure due to heavy administrative work, working overtime, overwhelming responsibility and shortage of healthcare workers [17,21,42]. Sharing of workload, support and team spirit were reported as motivating determinants and solutions to workload-related challenges [37]. A study in Tanzania reported healthcare workers in medium-sized (300–1000 patients per month) health facilities reported higher motivation than those in large-sized health facilities (>1000 patients per month) [19]. 

Training opportunities (or lack thereof) were among the top organizational level determinants of motivation among healthcare workers in East Africa [11,17,22,29,39,41,42,48,49]. Health workers reported the desire to engage in on-the-job learning, workshops, seminars, refresher courses and continuous training [20,44]. Training was viewed as an opportunity to access knowledge, experience and resources, to optimise skill utilisation and hope and to get more employment opportunities (for CHWs) or a promotion (for HCWs) [11,30,34,40,41,46]. However, some studies reported a lack of training opportunities or limited access to professional education due to lack of management coordination, favouritism and unfairness [20,42,43].

Shortage or lack of essential, medical supplies (such as gloves, syringes, cotton, medication) and equipment to facilitate medical treatment was reported as a demotivator [17,21,22,34,40,42,43]. Lack of initiative to recruit more professionals [42]; transport problems [17,33,34]; minimal job security, particularly in the private sector compared to the public [48]; lack of forms of identification (such as a license, badge and/or uniform), particularly among CHWs [33,44] and, problems with accommodation and housing [20,22] were some determinants of low motivation among colleagues [18]. 

Monetary support was a major motivating determinants among healthcare workers, which included requests for fair and timely payment of salaries [11,17,38,39,41,49], allowances [17,20], bonuses [33], compensation [33,40,42,50] and performance-based financing programs [45]. Researchers found that the higher healthcare workers’ salary, the more motivated they were to deliver high-quality healthcare services [32,35]. However, in a study in Rwanda, among the list of motivators, the monetary determinants ranked lower in the list compared to non-monetary determinants [36].

However, financially related demotivators were also reported, such as lack of remuneration, lack of compensation (financial or non-financial, such as airtime, bicycles, etc.), discrimination in allowances/per diems/salary and low salary [20,21]. The result was healthcare workers seeking other means of earning a living such as farming, working in the private sector or accepting informal payments in the form of bribes from patients [48].

Pertaining to schemes of service in the health system and health facilities, lack of job description or adherence to job description [32,42,48], and need for clear career progression or promotions procedures were among the determinants of healthcare workers’ motivation [18,21,22]; the need of supportive and better management was reported as a desired determinant of motivation, through enhanced coordination of and support of healthcare workers within the health systems [17,20,43]; and opportunities for family–career balance was reported as a motivating determinant in changing from practice to clinical research [18]. 

### 3.6. Methodological Quality 

The methodological quality of all 30 eligible studies included in this review were assessed. The tools used were Joanna Briggs Institute (JBI) Critical Appraisal Checklist tools [25] and the Mixed Methods Appraisal Tool (MMAT) [26]. Twenty-seven of the 30 eligible studies reviewed were assessed articles and of high quality. However, the remaining three studies included had a few unclear critical appraisal criteria, which were dealt with through statistical analysis such as multivariate regression analysis for the quantitative and mixed-methods studies. Following the assessment, the appraisal results according to the different study designs is reported in Table 4, Table 5 and Table 6.

## 4. Discussion

In this systematic review, we explored the determinants of healthcare workers’ motivation in the six East African Community (EAC) countries in the last decade (2009–2019). These countries are part of the Sub-Saharan Africa region, which has been described as having “severely weakened and under-resourced health systems” [21]. Sub-Saharan Africa has one of the greatest health-related challenges, with approximately 25% of the worldwide burden of disease in this region coupled with the shortage crisis of skilled healthcare professionals [3,49]. As countries work towards achieving Universal Health Coverage (UHC) and the Sustainable Development Goal 3: Health for all at all ages (SDG3), we need to devise effective health system strategies on how to work with, attract and retain the present health workforce. One of the ways is to motivate to join and most significantly stay in the health system. It is evident that the numbers, training and quality of human resources for health varies from country to country [1]; even with these similarities in determinants of healthcare workers’ motivation being discovered in this review among countries in the EAC. This review reports the individual, organizational/structural and societal determinants of healthcare workers’ motivation across the EAC states as reported by the health workforce.

Rudasingwa and colleagues stated that it is imperative that we integrate health workers’ definitions and perceptions of methods of enhancing quality of healthcare provision [45] and health workforce strengthening [10]. The motivating determinants of healthcare workers can also be viewed as facilitators of motivation, while the demotivating determinants can be viewed as barriers to their motivation. Both facilitators and barriers have been identified in this review at individual, organizational/structural and societal levels regarding the health workforce. Overall, motivating determinants were either individual-based while most demotivating determinants were organizational (health-facility-based) or structural (health-system-based). In our findings, while the individual determinants are mainly intrinsic in nature, the organizational and societal determinants are primarily extrinsic of the healthcare workers.

At the individual level, altruism is a prosocial behaviour that was the greatest motivating determinant among healthcare workers in the EAC. Altruism is a vital element of the medical discipline and has been described as a “core of competent health professionals’” [51] (p. 374). Medical altruism is rooted in the Hippocratic Oath, taken by healthcare professionals in the United States [51]. The desire to engage in prosocial behaviour involving saving lives, serving the community, sharing health-related knowledge and catalysing behavioural change within the community and public were some of the reasons for feeling a sense of responsibility to be altruistic [11,21,30,35,36,39,42,46]. According to the Theory-Based Model, altruism can be nurtured through education, practice and reinforcement [51]. 

Medical altruism has been found to affect the professionalism depicted in healthy clinical and patient relationships, resulting in better patient health outcomes and adherence and satisfaction with the quality of healthcare provided [47,50,51,52]. In the United Kingdom, medical altruism is a vital element of health workforce planning, for example in the UK National Health Service [52]. Examples of medical altruistic acts include working beyond contracted hours and providing additional information to their patients and their families, among other things [52]. The discovery of altruism or medical altruism among healthcare workers means managers and policymakers should pay attention to more than just the numbers. Focusing on the numbers only (quantity) can result in depletion of health among healthcare workers and cause demoralization, which are barriers to motivation [52].

In addition, a sense of usefulness within the community and in the health system after gaining knowledge and being able to use skills for the enhancement of healthy behaviour was echoed among respondents in various studies [30,34,47]. Three demotivating determinants at the individual level included perceived fear of contracting illnesses [42], being married [32] and work conscientiousness following a supervision intervention [37]. Health is a key concern among healthcare workers, particularly the risk and perceived fear of contracting illnesses’ [42]. A study in India reported risk of poor personal health being a demotivating factor, due to its detrimental effects on their ability to do their work [53]. As a result, health policymakers, managers and stakeholders ought to recognise the key role that feeling safe and protected through provision of protective gear plays and a safe working environment at large, have in reducing risk and fear of falling sick, thus increasing their motivation to work.

Work conscientiousness following a supervision intervention was found to decrease motivation among healthcare workers [37]. A possible explanation is the role of the type of leadership or supervision instigating autonomous motivation or controlled motivation regulations. This means the supervision intervention may have reduced the work conscientiousness among healthcare workers due to highly controlled motivation regulations. A study done among 547 nurses in Canada reported nurses being motivated by autonomous motivation as opposed to controlled motivation [54]. While autonomous motivation is self-regulated and intrinsic in nature, controlled motivation is the opposite and involves a lot of external control and complete lack of proactive independence in performing one’s work. Based on these finding, researchers encourage promotion of autonomous motivation over controlled motivation regulations to motivate and sustain healthcare workers [54].

The societal level, consisting of the clients (patients), community and family, was reported to play a significant role in the motivation of healthcare workers. The society was reported as playing a significant a role in positive reinforcement of healthcare workers’ motivation through appreciating, admiring, respecting, and recognising the work they do [11,20,30,33,34,36,40,41]. The desire to instigate healthy behavioural change within the community and the public was a major determinant of motivation across the health workforce in the EAC at the societal level.

However, the lack of appreciation, lack of recognition, dissatisfaction, insecurity and inability to adequately support their family, community and clients constituted the main demotivating societal-level determinants. These barriers to motivation at the societal level created a sense of helplessness [20] among healthcare professionals when receiving constant complaints or unexpected delays from the community or family of the clients occurred [34,43]. The presence of constantly escalating and irrational demands and expectations, such as the yearning for healthcare-related miracles of healthcare workers, instigated at times from the media, are barriers to medical altruism and motivation [52]. Instead, encouragement of honest and open-minded dialogue between clinician and patients which may be challenging at times for both parties needs to occur, as opposed to dialogue rooted in dishonesties [52]. Hence, it is paramount that we promote honesty, which could potentially result in an increased surge in medical altruism and motivation in healthcare workers. Health policymakers and stakeholders should recognise the integral role the community and society at large play in facilitating healthcare workers’ motivation as sources of reinforcement.

At the organizational and structural level, it is evident that greater attention to healthcare workers’ motivation is required [40]. The top three motivating determinants at the organizational/structural level were training opportunities [11,17,22,29,39,41,42,48,49], adequate monetary support suited for the living standards [11,17,38,39,41,49] and transformative leadership and supportive supervision [31,33,34,35,37]. Similar to this review, a study in India reported that regular training was reported as a motivating factor in India [53]. In Sweden, a study reported provision of remuneration as an important element of motivation, suggesting that monetary incentives be coupled with the quality of healthcare service delivery to patients [55]. These findings show that health policymakers and managers should aim at creating both monetary and non-monetary motivational packages for the health workforce [56].

Healthcare workers are motivated by transformative leaders who exhibit qualities such as idealised influence-behaviour, intellectual stimulation and inspirational motivation, not transactional or laissez-faire leaders [31]. Similarly, a study in Sweden emphasised the role of positive management, including provision of clear direction, non-hierarchical collaboration, clear communication and systematic empowerment geared towards motivating the healthcare workers [55]. In 2015, a study in Iran reported good and support manager and supervisors and having good working relations with co-workers being motivators of healthcare workers [57]. This emphasises the importance of having a healthy organizational social environment to motivate healthcare workers, which can enhance by strengthening management capacities and cohesive organizational culture [55,57]. These strategies could potentially result in a more supportive organization environment and curb the organizational-related barriers to healthcare workers’ motivation reported in various studies.

Barriers to motivation reported in studies included lack of or inadequate monetary support, favouritism, critical shortage of skilled healthcare professionals leading to heavy workload, and unrealistic expectations from management and government. Likewise, in Iran, unfair treatment, lack of appreciation and poor management were reported as demotivating factors [57], while in India heavy workload has been associated with job burnout [53]. Accepting the informal payment from patients resulted in deterioration in the quality and access of healthcare service delivery and individually, feelings of being overwhelmed and guilty were experienced by healthcare workers [48]. Therefore, it is essential to adequately compensate the health workforce to avoid compromising the universal access to healthcare, which is a right. 

The health workforce shortage crisis has adverse effects on the health systems and highlights the significance of motivation in quality of healthcare delivery and health outcomes among the already present health workforce [22,48]. The perpetual health workforce shortage is caused by the higher trained cadres such as physicians and nurses moving from public to private sectors or rural to urban migration, or brain drain, thus leaving the lower cadre staff and few cadre staff with a heavier load, yet the majority of the training opportunities are offered to the higher cadre staff, not the auxiliary staff. In developing countries, optimising the lower-level cadres and auxiliary staff by increasing training opportunities [1] to provide higher quality care from a knowledge- and skill-based stance will enhance health outcomes as a method of working around the shortage crisis. 

Healthcare workers have suggested that increased support, receiving constructive feedback, fair treatment and teamwork that involves sharing the workload are strategies that will enhance their motivation to provide quality healthcare while creating a sense of belongingness [11,37]. Additionally, autonomy has been reported to be a motivating determinant among healthcare workers. A Brazilian study among female dentists reported that their motivation to choose dentistry was the relative flexibility of practising the profession and being considerate of the entire well-being of their patients [58] whereas in Spain, doctors reported being intrinsically motivated and some financial controlling policies did weaken their intrinsic motivation [59]. 

An Indian study reported non-financial motivators reportedly need more attention in order to increase motivation among healthcare workers [53]. The findings of the studies in Spain [59] and India [48] are like the ones reported in this review. As much as both financial motivators are essential, non-motivators are equally important. Hence, redesigning flexible policies facilitating financial and non-financial motivation determinants among healthcare workers within health facilities and health sector at large need to be innovated and implemented.

Allowing the barriers to healthcare workers’ motivation to prevail results in their demotivation. Healthcare workers’ demotivation has an adverse ripple effect, from the reduced quality of care provided to poor patient outcomes, poor health outcomes and low performance at healthcare-worker, health-facility and health-system levels. Kok and colleagues stated that motivation is a critical determinant of performance [37] and thus is paramount in the functionality of health systems.

Even in resource-limited settings, it is essential to note that money cannot solve all the hardships in the health system [1], as much as it is a key facilitator. Therefore, non-monetary determinants of motivation identified can be used to create contextually relevant strategies to enhance the quality of healthcare services provision and strengthen health systems. Improvements and changes at the individual, organizational and societal levels are required based on the determinants of motivation and demotivation by the healthcare workers in the EAC health systems. This is vital to enhance the quality of care provided, health workforce strengthening [10], health system strengthening [8], achievement of Universal Health Coverage (UHC) [60] and the success if Sustainable Development Goal 3 (SDG:3) on health for all at all ages [61]. 

In the following recommendation section, stating the limitations of studies, possible considerations and solutions are geared towards enhancing health workforce motivation in the East African Community. 

### 4.1. Limitations in Studies Reviewed

Limitations reported in the various studies include limited sample size [18,37,46] and sample error related to the non-randomised techniques used to recruit participants such as convivence and purposive sampling [31,38,40,49]. Researchers stated that the limited sample size and related factors could potentially result in various biases, namely selection bias, social desirability bias, courtesy bias and response bias [29,30,31,36,39,40,49]. Due to limited or small sample size, researchers were aware of the limitations surrounding the possibility of the findings not being statistically generalizable to all healthcare workers [18,19,20,29,42,48,49]. However, due to similarities in the determinants reported among healthcare workers in multiple settings in the studies reviewed, the results should still be considered when creating informed strategies. Additionally cited limitations that may have affected the findings included the timing of the study [39,40], financial constraints [36] and use of a single measure [31]. 

### 4.2. Research Gaps and Recommendations for Further Research 

Based on the limitations and gaps cited in the literature reviewed, researchers recommended possible solutions. Researchers recommend future studies should use large sample sizes [22,34], preferably more heterogeneous samples, including all cadres—both support and professional healthcare workers [38]. For a broader picture, two studies recommended interviewing the healthcare workers who have left to know what would have motivated them to stay [34,41]. The need for more observational studies was identified due to the primarily subjective nature of most studies in this topic, as the majority were qualitative studies [36]. More quantitative studies using varied psychometrically tested measures in multiple settings, especially in rural areas, are needed [19,20,29,30,31]. The reason being tha the majority of the East African populations in all the six countries reside in the rural areas and are in more need of healthcare. 

Future studies should consider doing more correlational or causal studies on the relationship between the level of motivation and demographic factors [41], perceived performance [19] and effect of interventions on motivation [32,37]. More randomised studies are also needed to increase the possibility of generalisability of results; however, researchers also emphasis realistic, comprehensive approaches need to be taken when doing research and creating policies on this topic [22]. Additional studies in private health facilities would be essential for comprehensive and insightful analysis of determinants of healthcare workers’ motivation [29]. This is because the national health system includes both private and public healthcare facilities and workers.

A critical shortage of healthcare workers has been cited as a major challenge in the EAC, resulting in adverse ripple effects in the national health systems. Therefore, it is vital that we health policymakers, managers and ministries of health begin to recognise the importance of the already-present healthcare workers. Optimisation of the present health workforce will help improve the quality of healthcare delivery and strengthen health systems. Holistic programs [62] and context-specific training and supportive supervision of healthcare workers, especially the ones in lower cadres, are essential for the above to be achieved. The reason being that, in majority of Sub-Saharan health systems, lower-cadre and auxiliary healthcare workers are the majority compared to higher-cadre staff and are more likely to stay in the health system [1]. Therefore, as proven in this review, the desire for lower-cadre and auxiliary staff to be trained is high. The Ministries of Health in all the EAC member states should recognise this as an opportunity to work around the shortage of healthcare workers. Through empowering, training, rewarding and better remunerating the lower cadre staff for the work they already do due to the shortage, their motivation to remain in the health system and provide higher quality healthcare will prevail [11]. 

More studies regarding healthcare workers’ motivation in the EAC are needed to bridge knowledge gaps, some of which have been identified in this review. More literature has the potential of resulting in creation of tactically significant policies. Health-sector authorities should consider the inclusion of healthcare workers as stakeholders, decision-makers and policymakers in the strategic development and implementation initiatives [56]. This could result in more effective and relevant strategies, monitoring and evaluating of trends among healthcare workers [56]. These policies can facilitate health policymakers and implementers in the six EAC member states in decision-making processes regarding healthcare workers’ motivation and inform health-workforce-strengthening strategies.

## 5. Conclusions

The current systematic review reports both monetary and non-monetary determinants of healthcare workers’ motivation in the EAC between 2009 and 2019. It is evident that the individual, organizational/structural and societal determinants of healthcare workers’ motivation function interdependently, therefore representing the dynamism and intricacy involved in health workforce strengthening and health systems strengthening. This means holistic and contextually based understanding and approaches are needed to effectively solve the challenges surrounding the health workforce crisis in the EAC. Health policymakers, managers and implementers in the health systems could consider exploring the possibility of creating strategies based on the determinants of motivation synthesised and reported by healthcare workers. This is essential as nations work towards developing effective interventions geared towards attracting and retaining much-needed human resources for health within health systems in East Africa. Based on this review, research gaps in this area of focus have been highlighted for further exploration. This is important to guide the creation of improved strategies in the health systems development aimed at achieving UHC, SDG:3 among other health-related agendas.

## Figures and Tables

**Table 1:** Quantitative Studies.

Author(s), Year of Publication	Country	Main Objective	Sample Size	Main Research Findings
Leonard et al., (2010) [28]	United Republic of Tanzania	To examine the behaviour of 80 practitioners from the Arusha region of Tanzania for evidence of professionalism.	n = 80	Among 80 clinicians, approximately 20% were reported to have professionalism, an intrinsic motivator. Professionalism was characterised by having a small know–do gap and provision of high-quality diagnosis and high-quality communication with clients.
Momanyi et al., (2016) [29]	Republic of Kenya	To determine the influence of training on motivation among health workers in Narok County, Kenya.	n = 258	The health workers reported having an average level of motivation. On-the-job training significantly predicted general motivation among the health workers (p = 0.013).
Mpembeni et al., (2015) [30]	United Republic of Tanzania	To inform future scale-up, this study assessed motivation and satisfaction among these community health workers (CHWs).	n = 228	Motivational determinants explained 62% variance among the 228 CHWs, namely extrinsic stimuli, skill utilization, respect and hope, altruism and intrinsic needs. Statistically significant motivational determinants of CHWs included altruism (p = 0.0002) and intrinsic needs (p = 0.0384).
Musinguzi et al., (2018) [31]	Republic of Uganda	To examine the relationship between transformational, transactional and laissez-faire leadership styles and motivation, job satisfaction and teamwork of health workers in Uganda.	n = 564	Health workers were motivated more by health facility managers who used transformational leadership styles (p ≤ 0.05) and not those who used transactional and or laissez styles; transformational leadership skills had a positive impact on stimulating motivation.
Sato et al., (2017) [32]	United Republic of Tanzania	To measure three aspects of motivation: Management, Performance and Individual Aspects among health workers deployed in rural primary level government health facilities.	n = 263	Predictors of motivation among the 263 health workers were clear job descriptions (p ≤ 0.001) with regards to management and performance aspects; marital status was a predictor of the management aspect of motivation (p = 0.009); the number of years in their current profession was a predictor of management and performance aspects of motivation (<1year p = 0.043; >7year p = 0.042); and higher salary positively predicted the individual aspect of motivation as per salary scale (p = 0.029).
Siril et al., (2013) [19]	United Republic of Tanzania	To study healthcare workers (HCWs) stress, motivation, and perceived ability to meet patient needs were assessed in the United States President’s Emergency Program for AIDS Relief PEPFAR-supported urban HIV care and treatment clinics (CTCs) in Tanzania.	n = 279	Healthcare workers had significantly lower motivation than those in management (OR 0.26; 95% CI 0.10–0.73, p < 0.05) Healthcare workers in medium-sized sites (i.e., 300–1000 patients seen per month) experienced significantly higher motivation than those in large-sized sites (i.e., >1000 patients seen per month) (OR 2.00; 95% CI 1.05–3.82, p < 0.05).
Winn et al., (2018) [33]	Republic of Kenya	To identify factors related to the motivation and satisfaction of CHWs working in a malaria community case management CCM program in two sub-counties in Western Kenya.	n = 70	Influential determinants of motivation among the CHWs included altruistic personal desire to help the community (69%), gaining knowledge and experience (44%) responsibility within the community as a CHW (34%) appreciation and exposure within the community (30%), financial incentives (reimbursements, airtime and bonuses) (24%), and a good relationship with government supervisors 14%. Demotivating determinants of CHWs reported were transportation problems (21.3%) small clientele (14.2%) and inability to dispense medication to clientele (13.9%)

**Table 2:** Mixed Methods Studies.

Author(s), Year of Publication	Country	Main Objective	Sample Size	Main Research Findings
Brunie et al., (2014) [34]	Republic of Uganda	To examine factors related to CHW motivation and level of activity in 3 family planning programs in Uganda.	n = 226	Facilitating determinants of the CHWs motivation included acquiring new skills, social responsibility, enhanced status, helping the community, supportive supervision with helpful feedback, clients’ interest in family planning (programme) and hope for other opportunities such as future employment and advancements.
				Barriers to the CHWs motivation were lack of transport, stockouts of drugs and essential supplies, inability to support their own family from their job and complains from clients on family planning.
Chandler et al., (2009) [35]	United Republic of Tanzania	To evaluate factors affecting motivation, including reasons for varying levels of motivation, amongst clinicians in Tanzania.	n = 211	Quantitatively, among 177 clinicians’ higher salary was associated with intrinsic motivation and, was a prerequisite determinant for any other intervention to change motivation using non-financial ways.
				Qualitatively, among 34 clinicians, predominantly monetary-based determinants, the prestigious perception of status of being a healthcare professional and organizational social and physical environment were determinants of motivation.
Chin-Quee et al., (2016) [36]	Republic of Rwanda	To compare intervention and control districts and vis-à-vis CHWs’ work-related activities, their perceptions of workload manageability and reports of job satisfaction, motivation and service quality, as well as their clients’ reports of satisfaction and quality of care.	n = 400	The top three determinants of motivation among the CHWs’ in Rwanda were altruism, characterised by their desire to help their community; acquiring novel knowledge and skill; and getting and maintaining admirable professional status in the community. Receiving monetary and material goods was ranked low in the list of determinants of motivation among the community health workers. However, no statistically significant differences in determinants of the CHWs’ motivation were reported between the intervention and control groups.
Kok et al., (2018) [37]	Republic of Kenya	To assess whether this intervention influenced CHWs’ perceptions of supervision and CHW motivation over the period of 1 year after the implementation.	n = 74	Differences in the quantitative and qualitative results were evident regarding the effect of the supervision intervention on CHWs’ motivation. Qualitatively, CHWs reported the intervention enhancing their motivation due to its enhanced recognition, more support, increased knowledge, sharing the burden/workload and feeling of belongingness and team spirit. However, qualitatively, no statistically significant determinants were identified, but work conscientiousness significantly decreased from the baseline to midline p = 000,r = 0.29.
Ojakaa et al., (2014) [38]	Republic of Kenya	To investigate factors influencing motivation and retention of HCWs at primary health care facilities in three different settings in Kenya: the remote area of Turkana, the relatively accessible region of Machakos, and the disadvantaged informal urban settlement of Kibera in Nairobi.	n = 404	Among 404 healthcare workers, two statistically significant determinants of motivation were reported: manageability of workload had an odds ratio of 5.1 (CI = 2.1 to 12.0) and salary had an odds ratio of 13.5 (CI = 4.315 to 42.185)
				Qualitatively, facilitators of motivation included satisfaction with salary, job security, positive response from patients and good relations in the community. Demotivators were discrimination in training, poor housing quality, language barrier for non-locals, transport problems, lack of electricity, limited education choices, career stagnation, no allowances and no mentoring support from supervisors.
Sanou et al., (2016) [39]	Republic of Uganda	To investigate the factors influencing community health workers (CHWs) motivation and retention in health service delivery.	n = 134	Qualitatively identified determinants of motivation among CHWs comprised community recognition, status, regular training and provision of supplies.
				Quantitatively determinants of motivation included training opportunities (82.8%), opportunity to serve the community (79.9%), social knowledge or understanding (64.2%), supervision (41.0%), status in the community (53.0%), good working conditions (41.0%), supplementary income (24.6%), community support (farm work) (9.7%) and national benefits (0.7%).
Zinnen et al., (2012) [17]	United Republic of Tanzania	To contribute to empirical evidence on human resources for health motivation factors to assist policymakers in promoting effective and realistic interventions.	n = 285	Powerful motivators of the 285 human resources for health were primarily monetary, including salary and allowances.
				Non-monetary motivators varied, including the working equipment and conditions (environment), training and supervision, increased staff, better work environment, transport, good housing, enough drugs and supplies and better management.

**Table 3:** Qualitative Studies.

Author(s), Year of Publication	Country	Main Objective	Sample Size	Main Research Findings
Banek et al., (2015) [40]	Republic of Uganda	To understand the level of support available, and the capacity and motivation of community health workers to deliver these expanded services, we interviewed community medicine distributors (CMDs), who had been involved in the home-based management of fever (HBMF) programme in Tororo district, shortly before integrated community case management (ICCM) was adopted.	n = 100	The determinants of motivation involved an opportunity to be altruistic, gaining social status and recognition, creating future opportunities for employment and health-related knowledge gain.
				Demotivation sources were the community or government having unrealistic expectations, limited drugs and essential supplies such as gloves, poor supervision, and lack of compensation and respect as a result.
Daniels et al., (2013) [18]	Republic of Kenya	To present two distinct motivations for a clinical research career that informed women’s decision-making to pursue international training and describe two common steps in the pathway toward a clinical research career for women in Kenya.	n = 12	Two main determinants of motivation among the women medical doctors were professionally related motivators and attainment of family–career balance through engaging in clinical research. Demotivating determinants of the doctors included limited institutional capacity, low morale in the workplace and limited intellectual engagement.
Greenspan et al., (2013) [41]	United Republic of Tanzania	This study aimed to explore sources of community health workers motivation to inform programmes in Tanzania and similar contexts.	n = 20	Individual sources of motivation by CHWs comprised intrinsic desire to volunteer and support community, dedication to public service, desire for knowledge to help self and family and desire to educate the community.
				Organizational sources of motivation were monetary support, hope for future employment (job security), training tools for work and supervision.
				Family sources of motivation were moral, material and monetary support
				Community sources of motivation included recognition and encouragement through positive reception and acquiring fame in the community.
Kaye et al., (2010) [21]	Republic of Uganda	To assess the influence of this training experience on students’ willingness, readiness and competence to work in rural health facilities by surveying 60 recent graduates of Makerere University Faculty of Medicine, who completed their studies during the transition from traditional to problem-based learning (PBL) curriculum.	n = 60	Motivating determinants of the medical and nursing graduates to work in the rural areas included the desire to save lives (altruism), personal background (ease of communication) security, personal safety and opportunity for career advancement.
				Demotivating determinants of working in rural areas were inequitable and poor remuneration, high workload due to understaffing, no time for holidays, overwhelming responsibilities of clinical care, inadequate planning and heavy administrative work, low intellectual stimulation, inadequate supplies, equipment and supportive supervision, low access to continuing professional education, limited opportunities and discrimination in remuneration.
Mbilinyi et al., (2011) [42]	United Republic of Tanzania	To explore the challenges generated by human immunodeficiency virus (HIV) care and treatment and their impact on health worker motivation in Mbeya Region, Tanzania.	n = 30	Positive determinants of motivation (motivators) were mainly at the individual level, and the majority of the demotivators were at the organizational or health system structural level, with fewer demotivators being at the social-cultural environmental level.
Mbindyo et al., (2009) [11]	Republic of Kenya	To explore contextual influences on worker motivation, a factor that may modify the effect of an intervention aimed at changing clinical practices in Kenyan hospitals.	n = 185	Individual-level determinants of the healthcare workers’ motivation were altruism; appreciation; prestige from patients and family; professional attachment; sense of job security, especially in government; acquisition of career experience for career growth and development, and the challenge of meeting demands and expectations of patients.
				Organizational-level factors of motivation were resources and allocation, both human and non-human; relationship with colleagues and supervisors; fairness in treatment across cadres; incentives, both monetary and non-monetary; communication between hospital management and colleagues; recognition and appreciation, and commitment of managers to improving staff condition.
				Health system (structural)-level factors of motivation comprised schemes of service such as clarity of career progress, promotion, provision of allowances and salaries, career development possibilities and accessibility to training opportunities.
Mubyazi et al., (2012) [22]	United Republic of Tanzania	To describes the supply-related drivers of motivation and performance of health workers (HWs) in administering IPTp doses among other antenatal care (ANC) services delivered in public and private health facilities (HFs) in Tanzania, using a case study of Mkuranga and Mufindi districts.	n = 78	Key determinants of motivation/demotivation included poor working conditions of the health facilities (water, electricity, furniture); health worker shortage, leading to excess workload; shortage of essential drugs and supplies such as working gear and furniture. Private health facilities were more motivated because of better staff residences, better buildings, equipment, available clean water, electricity and cups for patients than public health facilities were. Public health facilities had more staff cadre such as clinical officers, nurses and midwives than private health facilities.
Mugo et al., (2018) [43]	Republic of South Sudan	To explore challenges and barriers confronted by maternal and child healthcare providers in delivering adequate quality health services to women during antenatal care visits, facility delivery and post-delivery care.	n = 18	Barriers to motivation in South Sudan included low salary, poor management and coordination, lack of supervision, shortage of healthcare workers, lack of training opportunities, lack of essential medical equipment, lack of security and absence of rewards (monetary e.g., bonuses or non-monetary incentives).
Prytherch et al., (2012) [20]	United Republic of Tanzania	To provide detailed understanding of the influences on the motivation, performance and job satisfaction of providers at rural, primary-level facilities were sought to inform a research project in its early stages.	n = 35	Key sources of motivation among the maternal and neonatal health (MNH) were community appreciation, perceived governmental and development support (per diems) and on-the-job learning (such as seminars and workshops).
				Prime sources of demotivation reported were mainly lack of fair compensation, unsupportive management, inflexible schedules, favouritism in promotions, uncertainty in transfer, poor security, poor health and safety, problems with accommodation and feelings of helplessness (due to lack of equipment and resources).
Ochieng et al., (2014) [44]	Republic of Kenya	To find out, from stakeholders’ perspectives, the type of tasks to be shifted to community health workers and the appropriate strategies to motivate and retain them.	n = 48	Strong motivators included close supportive supervision, means of identification, adequate resource allocation, continuous training and compensation.
Rudasingwa et al., (2017) [45]	Republic of Burundi	To what extent health workers are motivated and influenced by the Performance-Based Financing (PBF) scheme.	n = 36	Performance-based financing (PBF) motivated all the health workers and increased their teamwork and effort and enhanced their drive to change to implement best practice in their quality of service delivered.
Singh et al., (2016) [46]	Republic of Uganda	To understand whether full-time professional CHWs can potentially work with volunteers in the community to widen their reach and scope, and if so, what motivators might be of key importance to the community health volunteers (CHVs) remaining active in the field.	n = 81	Motivating determinants included desire to share health-related knowledge, relationship building, seeking health knowledge, being part of and seeing behavioural change within the community and the hope of gaining employment status.
Strachan et al., (2015) [47]	Republic of Uganda	The aim of this paper is to demonstrate how a behavioural theory, which accounts for the influence of group identification, in combination with data generated from qualitative interviews with CHWs and stakeholders, can be used to inform the design of interventions to improve CHW motivation, retention and performance in two settings—Uganda and Mozambique—with diverse, government-led HW programmes.	n = 87	The formative research in Uganda showed determinants of motivation were helping fellow community members; desire to provide proper healthcare services to the community; gaining their trust, respect and appreciation; learning; meeting new people; receiving validation and feedback from supervisors, and access to adequate resources such as drugs.
Stringhini et al., (2009) [48]	United Republic of Tanzania	To assess how informal earnings/payments might help boost health worker motivation and retention in Kibaha, Tanzania.	n = 64	Accepting of informal payment from patients to health workers had negative effects on health workers, access to and quality of health care services provided.
Takasugi et al., (2012) [49]	Republic of Kenya	This study sought to ascertain these motivational drivers.	n = 23	Both financial and non-financial motivational drivers were identified, including monetary and non-monetary rewards, specifically personal recognition, supportive supervision, personal development, training opportunities and good working conditions.
Witter et al., (2017) [50]	Republic of Uganda	To examine patterns in expressed motivation to join the profession across different cadres, based on 103 life history interviews conducted in northern Uganda, Sierra Leone, Cambodia, and Zimbabwe.	n = 26	Emerging determinants of motivation among the health workers were personal calling, professional status (admiration and respect), economically free tuition, perceived better pay, accommodation and transport, educational background, proximity to essential facilities and life events.

**Table 4:** Methodological quality of quantitative studies included.

Study	Critical Appraisal Checklist Item Numbers							
	1	2	3	4	5	6	7	8
Leonard et al., (2010) [28]	Y	Y	Y	Y	U	Y	Y	Y
Momanyi et al., (2016) [29]	Y	Y	Y	Y	U	Y	Y	Y
Mpembeni et al., (2015) [30]	Y	Y	Y	Y	U	Y	Y	Y
Musinguzi et al., (2018) [31]	Y	Y	Y	Y	U	Y	Y	Y
Sato et al., (2017) [32]	Y	Y	Y	Y	U	Y	Y	Y
Siril et al., (2013) [19]	Y	Y	Y	U	N	Y	Y	Y
Winn et al., (2018) [33]	Y	Y	Y	U	N	N	Y	Y

All the quantitative studies included were appraised using the Joanna Briggs Institute (JBI) checklist tools in accordance with the quantitative study design. Y: Yes; N: No; U: Unclear.

**Table 5:** Methodological quality of qualitative studies included.

Study	Critical Appraisal Checklist Item Numbers									
	1	2	3	4	5	6	7	8	9	10
Banek et al., (2015) [40]	Y	Y	Y	Y	Y	Y	Y	Y	Y	Y
Daniels et al., (2013) [18]	Y	Y	Y	Y	Y	Y	Y	Y	Y	Y
Greenspan et al., (2013) [41]	U	Y	Y	Y	Y	Y	Y	Y	N	Y
Kaye et al., (2010) [21]	U	Y	Y	Y	Y	N	Y	U	Y	Y
Mbilinyi et al., (2011) [42]	Y	Y	Y	Y	Y	Y	Y	Y	Y	Y
Mbindyo et al., (2009) [11]	Y	Y	Y	Y	Y	Y	Y	Y	Y	Y
Mubyazi et al., (2012) [22]	Y	Y	U	U	Y	N	Y	N	Y	Y
Mugo et al., (2018) [43]	U	Y	Y	Y	Y	Y	Y	Y	Y	Y
Prytherch et al., (2012) [20]	Y	Y	Y	Y	Y	Y	Y	Y	Y	Y
Ochieng et al., (2014) [44]]	Y	Y	Y	Y	Y	N	Y	Y	Y	Y
Rudasingwa et al., (2017) [45]	Y	Y	Y	Y	Y	Y	Y	Y	Y	Y
Singh et al., (2016) [46]	Y	Y	Y	Y	Y	Y	Y	Y	Y	Y
Strachan et al., (2015) [47]	Y	Y	Y	Y	Y	Y	Y	Y	Y	Y
Stringhini et al., (2009) [48]	Y	Y	Y	Y	Y	U	Y	Y	U	Y
Takasugi et al., (2012) [49]	Y	Y	Y	Y	Y	Y	Y	Y	Y	Y
Witter et al., (2017) [50]	Y	Y	Y	Y	Y	U	Y	Y	Y	Y

All the mixed methods studies included were appraised using the Mixed Methods Appraisal Tool (MMAT) version 2018. Y: Yes; N: No; C: Cannot tell; **-**: Not applicable.

**Table 6:** Methodological quality of mixed methods studies included.

Categories of Study Designs	Quality Checklist Item Numbers	Study						
		Brunie et al., (2014) [34]]	Chandler et al., (2009) [35]	Chin-Quee et al., (2016) [36]	Kok et al., (2018) [37]	Ojakaa et al., (2014) [38]	Sanou et al., (2016) [39]	Zinnen et al., (2012) [17]
Screening questions (for all types)	S1	Y	Y	Y	Y	Y	Y	Y
	S2	Y	Y	Y	Y	Y	Y	Y
1. Qualitative	1.1	Y	Y	Y	Y	Y	Y	Y
	1.2	Y	Y	Y	Y	Y	Y	Y
	1.3	Y	Y	C	Y	N	Y	Y
	1.4	Y	Y	N	Y	N	Y	Y
	1.5	Y	Y	C	Y	Y	Y	Y
2. Quantitative randomised controlled trials	2.1	−	−	−	C	−	−	−
	2.2	−	−	−	N	−	−	−
	2.3	−	−	−	N	−	−	−
	2.4	−	−	−	C	−	−	−
	2.5	−	−	−	Y	−	−	−
3. Quantitative non−randomised	3.1	−	−	−	−	−	−	−
	3.2	−	−	−	−	−	−	−
	3.3	−	−	−	−	−	−	−
	3.4	−	−	−	−	−	−	−
	3.5	−	−	−	−	−	−	−
4. Quantitative descriptive	4.1	Y	Y	Y	C	Y	C	Y
	4.2	Y	Y	Y	C	C	C	Y
	4.3	Y	Y	C	Y	Y	Y	Y
	4.4	Y	Y	N	Y	Y	C	Y
	4.5	Y	Y	Y	Y	Y	Y	Y
5. Mixed methods	4.1	Y	Y	N	Y	Y	Y	Y
	4.2	Y	Y	N	Y	C	Y	Y
	4.3	Y	Y	C	Y	C	Y	Y
	4.4	Y	Y	C	Y	Y	C	Y
	4.5	Y	Y	N	Y	Y	Y	Y

All the qualitative studies included were appraised using the JBI checklist tool in accordance with the qualitative study design. Y: Yes; N: No; U: Unclear.

## Abbreviations

**ANC**	Antenatal care
**CCM**	Community Case Management
**CHW**	Community Health Worker
**CHV**	Community Health Volunteer
**CMD**	Community Medicine Distributor
**CTC**	Care and Treatment Clinics
**EAC**	East African Community
**GHO**	Global Health Observatory
**HBMF**	Home-Based Management of Fever
**ICCM**	Integrated Community Case Management
**HCW**	Healthcare Worker
**HF**	Health Facility
**HIV**	Human Immunodeficiency Virus
**HW**	Health Worker
**MNH**	Maternal and Neonatal Health
**PBF**	Performance-Based Financing
**PBL**	Problem-Based Learning
**PEPFAR**	United States President’s Emergency Program for AIDS Relief
**PHC**	Primary Health Care
**PRISMA**	Preferred Reporting Items for Systematic Review and Meta-Analysis
**ROBIS**	Risk of Bias in Systematic Reviews
**SDG:3**	Sustainable Development Goal 3
**UHC**	Universal Health Coverage
**WHO**	World Health Organization
